# Model of translational cancer research in multiple myeloma

**DOI:** 10.1111/j.1349-7006.2012.02384.x

**Published:** 2012-08-17

**Authors:** Hiroshi Yasui, Tadao Ishida, Reo Maruyama, Masanori Nojima, Hiroshi Ikeda, Hiromu Suzuki, Toshiaki Hayashi, Yasuhisa Shinomura, Kohzoh Imai

**Affiliations:** 1 First Department of Internal Medicine, Sapporo Medical UniversitySapporo, Japan; 2 Department of Regional Health Care and Medicine, Sapporo Medical UniversitySapporo, Japan; 3 Department of Molecular Biology, Sapporo Medical UniversitySapporo, Japan; 4 Department of Public Health, Sapporo Medical UniversitySapporo, Japan; 5 Institute of Medical Science, The University of TokyoTokyo, Japan

## Abstract

Recently, intensive laboratory and preclinical studies have identified and validated therapeutic molecular targets in multiple myeloma (MM). The introduction of novel agents such as the proteasome inhibitor bortezomib and the immunomodulatory drugs thalidomide and lenalidomide, which were rapidly translated from preclinical studies at the Dana-Farber Cancer Institute into clinical trials, has changed the treatment paradigm and markedly extended overall survival; MM has therefore become a remarkable example of translational cancer research in new drug development. In this article, with the aim of determining the key factors underlying success in translational research, we focus on our studies of MM at Dana-Farber Cancer Institute as well as at our institutes. The identification of these key factors will help to promote translational cancer research not only in MM but also in other hematologic malignancies and solid tumors, to develop novel therapies, to overcome drug resistance, and to thereby improve the prognosis of cancer patients. (*Cancer Sci*, doi: 10.1111/j.1349-7006.2012.02384.x, 2012)

## Current approaches in multiple myeloma

Multiple myeloma (MM) is a neoplastic plasma cell disorder that is characterized by the clonal proliferation of malignant plasma cells in the bone marrow (BM), the presence of monoclonal immunoglobulin in the serum and/or urine in most cases, and associated organ dysfunction, including lytic bone lesions, compromised immunity, anemia, renal failure, and hypercalcemia.^1^ The combined use of melphalan and prednisone since the 1960s provided a median survival of 2–3 years for patients with MM. High-dose melphalan with autologous stem cell transplantation was established in the 1990s and this combination further increased the patient median survival to 3–4 years. However, MM was largely incurable, and therefore, novel biological treatment approaches were urgently required. In the last decade, the introduction of novel agents such as the proteasome inhibitor bortezomib and the immunomodulatory drugs (IMiDs) thalidomide and lenalidomide, which were rapidly translated from preclinical studies into clinical trials carried out by Anderson *et al*. at the Dana-Farber Cancer Institute (DFCI) of Harvard Medical School (Boston, MA, USA), has changed the treatment paradigm and markedly extended the overall survival.(1–5) Multiple myeloma has therefore become a remarkable example of translational cancer research in new drug development.(2) In this review, with the aim of determining the key factors underlying the success of translational research, we focused the several dozen studies we were engaged in at DFCI as well as our institutes.(2,3,6–8) This review consists of the following parts: (i) bases for translational research in oncology; (ii) novel targets and drugs; and (iii) biomarkers.

## Bases for translational research in oncology

Research in medical science is traditionally divided into two categories, basic and clinical research. Clinical research, as defined by the US National Institutes of Health (NIH), includes: patient-oriented research carried out on human subjects in which an investigator directly interacts with human subjects; epidemiological and behavioral studies; and research on outcomes and health services.(9) In contrast, basic research is carried out without considering practical ends and provides general knowledge.(9) To improve the prognosis of cancer patients, including those afflicted with MM, basic research and clinical research must be translated into practical applications. Translational research is a term that is used to describe the process by which the results of research carried out in the laboratory, in individuals (clinical), or in populations are used to develop new methods of diagnosis and treatment of a disease (clinical practice). The Translational Research Working Group of the National Cancer Institute (USA) also stated that the goal of translational research in oncology is to transform scientific discoveries arising from laboratory, clinical, or population studies into clinical applications to reduce cancer incidence, morbidity, and mortality.(10) Bridging the gap between basic research and clinical practice is a key factor for effective translational research. The NIH also concluded that barriers between clinical and basic research render translation of new knowledge to the clinic and back again to the laboratory bench difficult.(11) Therefore, translational research requires a close collaboration between basic scientists and clinical researchers, as well as between academia and industry.(2) Other key factors underlying the success of translational research include an understanding of the process of oncogenesis and the disease status as well as the identification of biological indicators for diagnosis, prognosis, and stratification.(12) In MM, the elucidation of tumor biology through clinical observations and genomic analyses accompanying the introduction of particular targeted drugs provide an example of bedside-back-to-bench research.(2) Ongoing translational research in MM includes genetic and epigenetic studies to evaluate myelomagenesis, identify targeted hallmarks of MM, and identify biomarkers to develop improved classification and personalized medicine; translational research also includes the development of novel therapies that target MM cells in the BM microenvironment.

## Novel targets and drugs

In 2011, Hanahan and Weinberg updated their proposition of the hallmarks of cancer that enable tumor cell growth and progression.(13) The proposed 10 hallmarks listed in Table 1 provide a framework for understanding cancer biology and therapeutic targets, and offer an effective way to organize and describe MM biology and therapeutic targets. For example, because nuclear factor κB (NFκB) activation in MM cells results in proliferative signaling and resistance to cell death, targeting the NFκB pathway is a promising therapeutic strategy in MM.(3,14) The concept of specific molecular targeting has been applied to the development of cancer therapies, and the two main approaches discussed here are the use of small-molecule agents and the use of therapeutic mAbs.(7) In Table 1, we list candidate small-molecule compounds that target proposed hallmarks in MM, most of which we and our colleagues have studied at DFCI and our institutes.(14–33) These small-molecule compounds that interfere with certain hallmarks of cancer are under development and are being investigated in clinical trials; in some cases, they have been approved for clinical use in the treatment of cancer, including MM.(2,8)

**Table 1:** Candidate small-molecule compounds targeting hallmarks in multiple myeloma

Hallmark	Candidate agent	Description	References
Sustainment of proliferative signaling	Perifosine	Inhibition of Akt	(15)
	Adaphostin	Abl cleavage	(16)
	CAL-101	Inhibition of PI3Kδ	(17)
	PKF115-584	Inhibition of β-catenin/TCF pathway	(18)
	SDX-308	Inhibition of β-catenin/TCF pathway	(19)
Evasion of growth suppressors	Seliciclib	Inhibition of cyclin-dependent kinase	(20)
Activation of invasion and metastasis	MLN3897	Inhibition of CCR1	(21)
Enabling replicative immortality	Imetelstat	Inhibition of telomerase	(22)
Induction of angiogenesis	Pazopanib	Inhibition of VEGFR	(23)
	IMiDs	Inhibtion of VEGF secretion	(24)
	Bortezomib	Inhibtion of VEGF secretion	(25)
Resistance to cell death	ABT-737	Inhibition of Bcl-2/Bcl-XL/Bcl-w	(26)
	R-etodolac	Upregulation of proapoptotic Mcl-1s	(27)
	MLN120B	Inhibition of IKKβ	(14)
	Bortezomib	Inhibition of NFκB	(3,28)
	IMiDs	Inhibtion of cytokine secretion	(3,29)
Prevention of immune destruction	IMiDs	Inhibtion of IL-2 secretion	(30,60)
Deregulation of cellular energetics	Cerulenin	Inhibition of fatty-acid synthase	(31)
Genome instability and mutation	ABT-888	Inhibition of PARP	(32)
Tumor-promoting inflammation	MLN120B	Inhibition of IKKβ	(14)
	BIRB 796	Inhibition of p38 MAPK	(33)
	IMiDs	Inhibtion of cytokine secretion	(3,29)

CCR1, chemokine (c-c motif) receptor-1; IKKβ, IKB kinase β; IL-2, interleukin-2; IMiDs, immunomodulatory drugs; NFκB, nuclear factor κB; PARP, poly(ADP-ribose) polymerase; VEGF, vascular endothelial growth factor; VEGFR, vascular endothelial growth factor receptor.

**Fig. 1:**
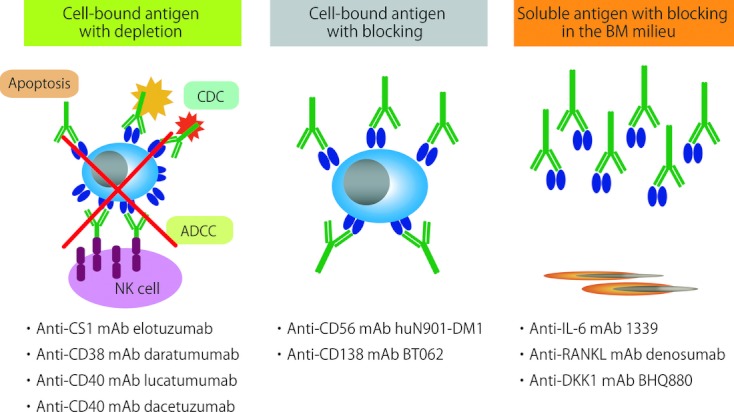
Classification of therapeutic antibodies based on their mechanisms of action. Class I antibodies recognize and bind to cell-bound antigens. The Fc effector functions are part of the mechanism of action of antibodies. Class II antibodies also recognize and bind to cell-bound antigens but their proposed mechanism of action does not involve Fc effector functions. Class III antibodies bind to and neutralize soluble antigens, and their mechanism of action often involves blocking the soluble ligand from binding to its receptor. ADCC, antibody-dependent cell-mediated cytotoxicity; BM, bone marrow; CDC, complement-dependent cytotoxicity; NK, natural killer.

In addition, mAb-based therapies for MM are currently being developed.(7,8,34) Figure 1 presents a list of therapeutic antibodies in MM, which can be divided into three classes based on their mechanisms of action.(35) Class I antibodies recognize and bind to cell-bound antigens to kill target cells through crystallizable fragment-mediated effector functions, including antibody-dependent cell-mediated cytotoxicity (ADCC), complement-dependent cytotoxicity, and antibody-dependent apoptosis.(36) The anti-CD38 mAb daratumumab(37) and anti-CD40 mAbs lucatumumab and dacetuzumab(38) are currently in preclinical and clinical development for the treatment of MM.(7,8,34) Our studies show that IMiDs can augment ADCC triggered by mAbs including elotuzumab,(30,36) and this finding provides the rationale for a combination clinical trial.

Class II antibodies also recognize cell-bound antigens, but their proposed mechanism of action does not involve crystallizable fragment effector functions. The main functions of this class of antibodies are to block ligand–receptor interactions and to act as immunoconjugates to convey intracellular toxins or radioactive isotopes. In MM research, novel mAb–maytansinoid immunoconjugates (huN901-DM1, which binds to CD56,(39) and BT062, which binds to CD138(40)) are being developed.

Class III antibodies bind to and neutralize soluble antigens, and their mechanism of action often involves blocking of the soluble ligand from binding to its receptor. A human mAb 1339 targeting interleukin-6 (IL-6) is being developed for MM.(41) The targeting of soluble ligands that affect bone biology by using mAbs such as the anti-RANKL mAb denosumab(42) and the anti-DKK1 mAb BHQ880(43) is promising, not only for preserving bone integrity but also for treating MM.(34) In January 2012, denosumab was approved in Japan for the treatment of bone complications caused by MM.(42)

The biological diversity of tumor cells within the BM microenvironment may influence the number of targets for MM therapies.(2,3,6,8) Candidate agents and approved novel drugs can show significant antitumor activity in MM *in vitro*, but treatment with single agents may not provide sufficient clinical efficacy because of drug resistance. Successful treatments can thus be achieved using other hallmarks and by addressing drug resistance. Therefore, we envisage that the use of functionally multitargeting drugs, as listed in Table 2, will provide effective MM therapies.(2,8)

**Table 2:** Functionally multitargeting agents in multiple myeloma

Classification	Candidate agent	Description	References
Proteasome inhibitor	Bortezomib	Inhibition of chymotryptic-like and caspase-like activities	(28,44)
	MLN9708	Inhibition of chymotryptic-like and caspase-like activities	(46)
	Carfilzomib	Inhibition of chymotryptic-like activity	(47)
	Marizomib (NPI-0052)	Inhibition of chymotryptic-like, trypsin-like, and caspase-like activities	(48)
HDAC inhibitor	Vorinostat (SAHA)	Pan-HDAC inhibitor	(49)
	Panobinostat (LBH-589)	Pan-HDAC inhibitor	(50)
	Tubacin	HDAC6 selective inhibitor	(51)
	ACY-1215	HDAC6 selective inhibitor	(52)
HSP inhibitor	IPI-504 (tanespimycin)	Inhibition of hsp90	(54)
	SNX-2112	Inhibition of hsp90	(55)
Drugs influencing lysophospholipid signaling	Perifosine	Inhibition of Akt	(15)
	FTY720	Sphingosine 1-phosphate agonist	(57)
	LPAATβ inhibitor	Inhibition of LPAATβ	(58)
IMiD	Thalidomide	Inhibtion of cytokine secretion	(29,59)
	Lenalidomide (CC-5013)	Inhibtion of cytokine secretion; induction of apoptosis	(29)
	Pomalidomide (CC-4047)	Inhibtion of cytokine secretion; induction of apoptosis	(29,59)
DNA methyltransferase inhibitor	Decitabine	DNA methyltransferase inhibitor	(69)

HDAC, histone deacetylase; HSP, heat shock protein; IMiDs, immunomodulatory drugs; LPAATβ, lysophosphatidic acid acyltransferase β.

### Proteasome inhibitors

The ubiquitin proteasome pathway regulates the turnover of many intracellular proteins that are tagged with multiple ubiquitin molecules for transport to the 26S proteasome for subsequent degradation. Bortezomib is a prototype 26S proteasome inhibitor that selectively binds to and reversibly inhibits chymotrypsin-like and caspase-like activity.(28,44) In 2001, Hideshima *et al*. reported that bortezomib regulates cell cycle proteins in MM cells and targets intrinsic and extrinsic apoptotic pathways. It also inhibits the secretion of IL-6 and vascular endothelial growth factor triggered by the binding of MM cells to BM stromal cells and inhibits BM angiogenesis by exerting a direct inhibitory effect on endothelial cells.(25) Bortezomib has undergone a remarkable transition from bench to bedside; a phase II study of bortezomib revealed a 35% response rate with manageable toxicity, and bortezomib was then approved by the US Food and Drug Administration (FDA) for the treatment of relapsed/refractory MM in 2003.(45)

Recently, the orally active agent MLN9708,(46) carfilzomib which selectively inhibits chymotrypsin-like activity,(47) and the broad-based proteasome inhibitor marizomib(48) have been developed in preclinical and clinical studies. Marizomib inhibits chymotrypsin-like, trypsin-like, and caspase-like activity and induces apoptosis in MM cells resistant to conventional agents and bortezomib.(48)

### Histone deacetylase inhibitors

Histone deacetylases (HDACs) are enzymes involved in the remodeling of chromatin and play a key role in the epigenetic regulation of gene expression, which ultimately mediates cellular differentiation and survival.(44) The combination of bortezomib with HDAC inhibitors has yielded promising results in preclinical MM models and will thus be applied to clinical trials.

The HDAC inhibitors can be divided into two groups: non-selective pan-HDAC inhibitors such as vorinostat(49) and panobinostat(50) that predominately target Class I (HDAC1, HDAC2, and HDAC3) and Class IIb (HDAC6) HDACs; and Class I HDAC inhibitors such as romidepsin and entinostat that target only Class I HDACs.(44) Although the mechanism underlying the synergistic activity of HDAC inhibitors with bortezomib is not fully understood, it may involve the role played by HDAC6 in the aggresomal degradation of ubiquitinated proteins.(44,51) The preclinical activity of a novel HDAC6 inhibitor, ACY-1215, alone and in combination with bortezomib, was recently reported(52) and transformed into a clinical study.

### Heat-shock protein inhibitors

Heat-shock proteins (HSPs) constitute a class of molecular chaperones that, under normal conditions, facilitate protein folding and regulate the turnover of proteins involved in cell growth and survival. Under conditions of environmental stress, HSP expression increases as an adaptive means to maintain cell homeostasis and enhance cell survival. Because bortezomib induces the expression of stress response-related proteins such as hsp27, hsp70, and hsp90, these proteins are molecular targets for overcoming bortezomib resistance.(53) Inhibition of p38MAPK, which is an upstream molecule of hsp27, enhances the cytotoxicity of bortezomib in MM cells, thereby providing evidence that hsp27 confers bortezomib resistance.(33) Hsp90 inhibitors such as 17-AAG (tanespimycin),(53) IPI-504 (retaspimycin hydrochloride, which is a water-soluble analog of tanespimycin),(54) and SNX-2112(55) enhance bortezomib-induced cytotoxicity in preclinical models. IPI-504 has been translated into a clinical study in MM.(56)

### Drugs influencing lysophospholipid signaling

We evaluated several drugs that influence lysophospholipid signaling, such as the sphingosine 1-phosphate analogue FTY720,(57) an LPAATβ inhibitor,(58) and perifosine.(15) Perifosine, which is an alkyl-phosphocholine compound, has been shown to inhibit Akt activation without affecting the activity of PI3K or phosphoinositide-dependent kinase 1. Because perifosine inhibits the Akt activation triggered by bortezomib to enhance MM cytotoxicity *in vitro*, combined therapy with bortezomib and drugs that inhibit Akt signaling is promising. Perifosine in combination with bortezomib is being evaluated in clinical trials.

### Immunomodulatory drugs

The IMiDs have several anti-MM effects, including direct cytotoxicity, inhibition of angiogenesis, and induction of tumor immunity, and provide a remarkable example of translational cancer research in MM. In 2000, Hideshima *et al*.(29) reported the mechanism of anti-MM activity of the IMiDs lenalidomide (IMiD3, CC5013) and pomalidomide (IMiD1, CC4047), which potently induce apoptosis or growth arrest in MM cells. The IMiDs also reduce the secretion of IL-6 and vascular endothelial growth factor triggered by the binding of MM cells to BM stromal cells, and they inhibit angiogenesis.(59) Lenalidomide was rapidly applied to clinical trials and was approved by the FDA in 06 for use in patients who have received prior therapy.(1) The IMiDs also stimulate a T cell co-stimulatory mechanism to induce IL-2 expression and T-cell proliferation.(60) Moreover, IMiDs induce natural killer (NK) cell-mediated cytotoxicity because the proliferation and ADCC of NK cells are induced by IL-2 production.(30) These data provide the cellular and molecular basis for the use of IMiDs as an adjuvant in immunotherapeutic treatment strategies for MM.

## Biomarkers

A biomarker, as defined by NIH, is a characteristic that is objectively measured and evaluated as an indicator of normal biologic processes, pathogenic processes, or pharmacologic responses to a therapeutic intervention. Biomarkers can be classified based on their application, such as diagnostic biomarkers, biomarkers for the staging of diseases, biomarkers for disease prognosis, and biomarkers for monitoring the clinical response to an intervention. Genetic heterogeneity has been indicated in MM, and has important implications for tumor pathogenesis, prognosis, and treatment. Importantly, cytogenetic aberrations, including the non-hyperdiploid, cytogenetically detected chromosomal 13q deletion as well as *t*(4,14), *t*(14,16), 1q gain, and del(17p), as detected by FISH, are indicators of high-risk MM associated with a poor outcome.(1) Novel therapies such as bortezomib can overcome, at least in part, the adverse outcome conferred by these abnormalities.(2) However, there has been much less progress in the development of predictive biomarkers for specific treatments.(12) To identify biomarkers to predict the effect of particular targeted therapies, appropriate clinical trial designs are necessary. Phase I studies are needed to establish that the drug inhibits the targeted pathway in the tumor. Phase II studies are required to obtain data for determining predictive biomarkers that identify patients whose tumors are driven by the inhibition of the target molecule so that therapy-specific diagnostic tests can be developed for phase III trials. Because some novel drugs in development in MM have specific molecular targets, the identification of biomarkers that also define drug sensitivity is a promising therapeutic strategy. Examples include the use of PI3K inhibitors in patients who show PI3K activation and IκB inhibitors in patients who show activation of the NFκB pathway. Efforts to examine patient samples by genetic, cytogenetic, and epigenetic methods are important to identify biomarkers to improve patient classification and, if possible, introduce personalized therapy for MM.(2) In this review, we focus on genetic studies that have recently been facilitated by next-generation sequencing technologies, as well as focus on DNA methylation studies that we are engaged in at our institute.

### Genetics in MM

Major tumor-genome sequencing projects have been undertaken to identify the numerous genes mutated in cancer.(61) However, the key steps in oncogenesis in human tumors remain unclear. In MM, genomic studies are currently being carried out for the definition of heterogeneity, new target discovery, and development of personalized therapy. The analysis of somatic mutations by sequencing of the tumor genomes in 38 MM cases revealed that the mutated genes involved in NFκB activation, protein homeostasis, and histone methylation are consistent with MM biology.(62) Moreover, activating mutations of *BRAF* were observed in 4% of patients; this finding has immediate clinical translational implications for the use of BRAF inhibitors. It is important to distinguish the driver mutations from the passenger mutations; a driver mutation is defined as a mutation that is causally implicated in oncogenesis, whereas a passenger mutation is defined as a mutation that has no effect on the fitness of a clone but is present in the same genome with a driver mutation.(61) The existence of several driver mutations in individual cancer is consistent with the hallmarks of cancer.(13)

### DNA methylation in MM

DNA methylation, which occurs in cytosine bases located 5′ to a guanine in which the cytosine–guanine pairs are known as CpG or CG dinucleotides, is catalyzed by DNA methyltransferases (DNMT1, DNMT3A, and DNMT3B).(63) Various cancers are characterized by promoter hypermethylation and consequent epigenetic silencing of multiple genes, and this process can be reversed during DNA synthesis, which renders it a potential therapeutic target.(63) The DNA methyltransferase inhibitors azacitidine and decitabine (5-aza-2′-deoxycytidine) have remarkable activity in the treatment of myelodysplastic syndrome (MDS), and both were approved by the FDA for the treatment of patients with MDS.(8) We and others studied DNA methylation in MM and identified certain key genes, including *RAS*, *dexamethasone-induced 1* (*RASD1*), listed in Table 3.(64–69) Interestingly, MM cells that showed methylation of RASD1 were resistant to dexamethasone, and treatment with decitabine restored RASD1 expression and enhanced the cytotoxicity of dexamethasone in tumor cells. The methylation levels of RASD1 in clinical samples were elevated after repeated chemotherapy, including therapy with dexamethasone. The goal of our ongoing studies is to define RASD1 methylation as a predictive indicator of steroid resistance in MM. Our findings suggest that epigenetic gene silencing is involved in MM progression and drug resistance, and DNA methylation can therefore be a potential biomarker for MM. We are also engaging in genome-wide methylation analyses to determine the molecular mechanisms underlying MM, including oncogenesis, drug resistance, and the heterogeneity of genetic, cytogenetic, and epigenetic aberrations, thereby identifying biomarkers in MM.

**Table 3:** Genes epigenetically silenced in multiple myeloma

Gene	Chromosomal location	Function	References
*CDKN2A* (p16^INK4A^)	9p21.3	Inhibition of cyclin-dependent kinase	(64)
*CDKN2B* (p15^INK4B^)	9p21.3	Inhibition of cyclin-dependent kinase	(64)
*CHFR*	12q24.33	Mitotic checkpoint	(65)
*RASSF1A*	3p21.31	Inhibition of Ras signaling	(66)
*DAPK1*	9q21.33	Induction of programmed cell death	(67)
*BNIP3*	10q26.3	Induction of apoptosis	(68)
*RASD1*	17p11.2	Modulation of coregulator activity of NONO	(69)

## Perspectives and conclusions

Ongoing translational cancer studies in MM include: genetic and epigenetic studies to evaluate myelomagenesis, identify targeted hallmarks of MM, and develop improved classification and personalized medicine; the development of next-generation novel therapies targeting MM cells in the BM milieu; and the development of rationally based combination therapies.(2,3,8) To date, many preclinical studies have hinted at the myriad of pathways that can be targeted for a synergistic and multitargeted approach. To identify these areas of molecular synergism, close collaboration between basic researchers and clinical staff is critical. These efforts will help to develop novel therapies, overcome drug resistance, and improve the prognosis of patients with MM.
